# Emergency Management of Intoxication‐Type Inherited Metabolic Disorders

**DOI:** 10.1002/jimd.70007

**Published:** 2025-02-14

**Authors:** J. Dexter Tarr, Andrew A. M. Morris

**Affiliations:** ^1^ Willink Metabolic Unit, Genomic Medicine St Mary's Hospital Manchester UK; ^2^ Willink Metabolic Unit, Genomic Medicine, St Mary's Hospital and Faculty of Biology, Medicine and Health University of Manchester Manchester UK

**Keywords:** cardiomyopathy, encephalopathy, hyperammonaemia, liver failure, rhabdomyolysis, seizures

## Abstract

In many intoxication‐type inherited metabolic disorders, the accumulation of the toxic chemical can cause acute life‐threatening emergencies. Sometimes this is the inevitable consequence of a severe metabolic defect, but it is often triggered by catabolism. In this article, we consider the acute management when these conditions cause encephalopathy, seizures, stroke‐like episodes, thromboses, liver failure, cardiac failure, arrhythmias and rhabdomyolysis. Treatment is available for most intoxication‐type disorders, though it is seldom entirely satisfactory. The emergency management involves general measures for the immediate problem (such as liver failure, thrombosis or an arrhythmia) and specific treatment for the metabolic disorder. The latter usually aims to reduce the accumulation of the toxic small molecule. Often this involves preventing or reversing catabolism. Sometimes the formation of the toxic chemical can be reduced by removing dietary precursors, by diverting precursors to alternative pathways, or by inhibiting an earlier step in the affected pathway. Another strategy is to remove the toxic chemical by binding it to a drug or by extracorporeal blood purification. Occasionally, the block in the pathway can be ameliorated and some disorders, specific treatment may prevent the consequences of the accumulating chemical. Despite all these treatment strategies, outcomes are often disappointing, particularly if an intoxication disorder first presents as an emergency. Newborn screening has greatly improved the prognosis for some disorders. For others, outcomes can only be improved by earlier recognition and treatment.

## 1 Introduction

Intoxication‐type inherited metabolic disorders represent one of the simplified groupings of inherited metabolic disease, along with disorders resulting from small molecule deficiency, defects of energy metabolism and complex molecule disorders. The clinical features result from the accumulation of small molecules proximal to a ‘block’ in their metabolism [1]. Molecules that are harmless or even essential at low concentrations are often toxic at high levels. Intoxication‐type metabolic disorders may present with acute or chronic symptoms; most are treatable, and acute presentations require emergency management.

**FIGURE 1:**
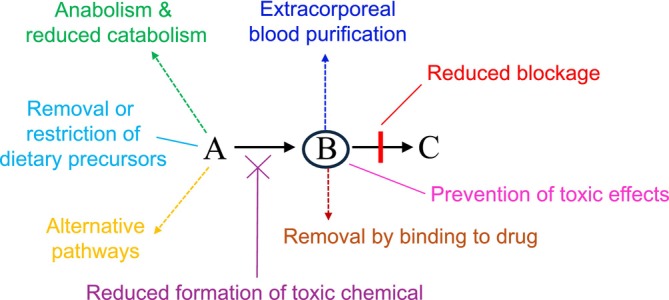
Schematic diagram showing potential treatment strategies for intoxication‐type metabolic disorders. A, B and C represent molecules in a metabolic pathway, in which an inborn error impairs the conversion of B to C.

There are many potential ways to treat intoxication‐type disorders, as illustrated in Figure 1. The best management is generally to prevent the accumulation of the toxic chemical by reducing flux through the affected pathway. This can often be achieved by reducing catabolism and promoting anabolism. Alternatively, it may involve removing dietary precursors of the toxic chemical, such as galactose in galactosaemia. The formation of the toxic chemical can sometimes be reduced by diverting precursors to alternative pathways (as nitrogen scavengers do in urea cycle disorders) or by inhibiting an earlier step in the affected pathway (as nitisinone does in tyrosinaemia type 1). Occasionally, the block in the pathway can be improved; for emergency management, this is mostly the correction of a secondary block, such as the correction of urea cycle inhibition in organic acidaemias using carglumic acid. Sometimes the toxic chemical is removed by binding to a drug, such as a copper chelator, or by extracorporeal blood purification. Finally, specific treatment may prevent the consequences of the accumulating chemical, as pyridoxine does in antiquitin deficiency.

Acute problems in intoxication‐type disorders include hypoglycaemia, acidosis, dehydration, encephalopathy, seizures, stroke‐like episodes, thromboses, liver failure, cardiac failure, arrhythmias and rhabdomyolysis. Obviously, the management (and investigations, if the diagnosis is unknown) depends on the clinical presentation, but we will start with some general considerations.

## 2 Initial Assessment and Investigation

Management of acutely ill patients starts with the assessment of the airway, breathing and circulation. Ventilation may be needed, particularly in neonates, and these patients often require intravenous fluids to correct dehydration. Initial investigations in patients who may have an intoxication disorder should include the measurement of the blood glucose and ammonia concentrations and acid–base status. Hypoglycaemia should be corrected with an intravenous bolus of glucose followed by an infusion (preferably after taking samples if the diagnosis is unknown). An infusion of sodium bicarbonate (suitably diluted) should be considered if severe metabolic acidosis (pH < 7.1 and base deficit > 10 mmol/L) persists after initial resuscitation, for example, in organic acidaemias and glutathione synthetase deficiency. In intoxication‐type disorders, acidosis is less likely to be corrected by other measures than in other conditions (such as shock, diabetic ketoacidosis or defects of gluconeogenesis).

In some patients presenting acutely, the diagnosis is already known. This allows prompt management to minimize problems. The expansion of newborn screening over recent decades has improved the outcomes for many diseases [2].

Urgent investigations are needed if the diagnosis is unknown. As intoxication‐type metabolic disorders are caused by the accumulation of small molecules, they can usually be diagnosed by biochemical tests, such as plasma amino acids, total homocysteine, acylcarnitines, urine organic acids and erythrocyte galactose‐1‐phosphate uridyl transferase, with results within a few hours. Genetic testing is generally slower, but advances mean that some centers can obtain whole genome sequencing results within 3 days [3]. This is most valuable for disorders where no biochemical test is available, but it may occasionally identify an unsuspected intoxication disorder. Genetic tests must not, however, displace metabolic investigations, which are better for disorders requiring acute treatment.

## 3 Preventing Catabolism and Promoting Anabolism

Acute presentations are usually triggered by increased flux through the affected pathway; this may be due to changes in diet or growth, but it is usually due to catabolism caused by an infection, fasting or trauma. Many disorders present in the neonatal period after a symptom‐free interval lasting hours or days; these patients have little residual flux through the pathway, and accumulation of the toxic chemical is inevitable, but it is worsened by the catabolism that always follows birth. Patients with greater residual activity of the affected enzyme or transporter often present later in childhood or even as adults, when catabolism leads to ‘decompensation’. Causes of catabolism should, therefore, be sought and treated (e.g., with antibiotics) in any patient presenting acutely with metabolic decompensation.

Anabolism can be promoted by giving glucose, which causes the secretion of insulin and reduces the secretion of stress hormones, such as cortisol. Lipids can also be given to most patients except those with fatty acid oxidation disorders. Severely ill patients will require intravenous management, which should provide at least the age‐related energy requirement. An insulin infusion allows more glucose to be given, promoting anabolism without causing hyperglycaemia, the infusion rate being adjusted according to the glucose concentration. Growth hormone can also promote anabolism in intoxication‐type disorders [4] but benefit has not been shown in the emergency situation.

Initially, when patients may be catabolic, it is appropriate to stop their normal diet. Dietary protein might, for example, increase the toxic chemical accumulating in a defect of amino acid degradation. It is important, however, to reintroduce the diet soon, preferably within 24 h [5], as the lack of nutrients will otherwise worsen catabolism.

Following diagnosis, families can often prevent decompensation in these disorders by starting regular drinks containing maltodextrin (±lipid) at home at the first sign of an illness. This is sometimes referred to as an ‘Emergency Regimen’ and it avoids the delay that is inevitable when going to hospital; moreover, higher concentrations of glucose can be given enterally than through a peripheral intravenous cannula.

## 4 Prevention of Encephalopathy in Maple Syrup Urine Disease and Glutaric Aciduria Type I

In some disorders, extra measures are taken acutely to reduce catabolism through the affected pathway. Specific amino acid mixtures are added to maltodextrin in the emergency regimens for maple syrup urine disease (MSUD) and glutaric aciduria type I (GA 1). These are both disorders of amino acid degradation in which catabolism can lead to acute encephalopathy with few systemic features but a risk of permanent brain damage. Newborn screening is now widely undertaken for both disorders, allowing the use of the Emergency Regimen to prevent decompensation and neurological injury.

*MSUD* is caused by deficiency of the branched‐chain 2‐ketoacid dehydrogenase (BCKD) complex, which catalyzes the second step in the degradation of the three branched‐chain amino acids (BCAA). As the initial transamination step is reversible, this leads to the accumulation of leucine, isoleucine and valine, as well as the corresponding ketoacids. BCAA excretion is limited by reabsorption from the renal tubules, so the best way to reduce BCAA levels is by incorporating them into protein synthesis. The effectiveness of this is enhanced by leucine's anabolic effects: raised plasma levels reduce protein breakdown [6], promote protein synthesis [7] and increase insulin secretion [8].

In the long‐term, MSUD patients are managed with a low‐BCAA diet, that is, small, measured amounts of natural protein and BCAA‐free amino acid supplements. To minimise BCAA rises during illnesses, BCAA‐free amino acids are added to maltodextrin in the Emergency Regimen to promote protein synthesis. As leucine is the most toxic BCAA, some isoleucine and valine are generally also added to the Emergency Regimen, but plasma amino acid analysis is essential for fine‐tuning during recovery [9]. If MSUD patients vomit their emergency drinks, they can be given glucose, fluids and electrolytes intravenously and a concentrated BCAA‐free amino acid mixture slowly through a nasogastric tube. Though intravenous BCAA‐free amino acid mixtures have been used [10], they are not widely available [11]. Extracorporeal blood purification is needed if patients are profoundly encephalopathic, with leucine concentrations considerably above 1000 μmol/L. These procedures are needed most frequently when patients are first diagnosed, and they are considered below in the section on hyperammonaemia. Intensive management of any cerebral oedema is also needed [9]. Unfortunately, there may be lasting intellectual impairment [11], which correlates with the peak plasma leucine concentration [12].

High‐dose thiamine is sometimes given to MSUD patients as there is a thiamine‐responsive form [13] but this is extremely rare. Sodium phenylbutyrate has been shown to reduce leucine levels in patients with intermediate MSUD, apparently by inducing increased BCKD activity [14]. Orphan drug designation has been given by the FDA for this indication, and benefit has been reported in a cohort of acutely ill patients when there was no capacity for haemofiltration [15] but further trials are needed to establish its role in management [16]. Other developments relate to long‐term rather than emergency management. Liver transplantation for MSUD is well established [17], whilst oral enzyme replacement therapy [18] and gene therapy [19, 20] are being developed.

*GA 1* is caused by a deficiency of glutaryl‐CoA dehydrogenase (GCDH), which is involved in the metabolism of lysine, hydroxylysine and tryptophan. Without treatment, most patients present with acute encephalopathy between 3 months and 3 years of age, usually following a febrile infection. Subsequently, they have a severe movement disorder with dystonia, orofacial dyskinesia and truncal hypotonia. A few patients have a gradual onset of dystonia [21]. Damage to the basal ganglia is thought to be caused by glutaric and 3‐hydroxyglutaric acids and glutaryl‐CoA, which accumulate within the brain because the blood–brain barrier has a low permeability for dicarboxylic acids. The risk of neurological damage is greatly reduced by dietary management. When well, patients consume small, measured amounts of natural protein and amino acid supplements that are lysine‐free and low in tryptophan [22]. During illness, patients are given an Emergency Regimen containing maltodextrin and the lysine‐free amino acid mixture. This lowers the lysine concentration relative to other amino acids, including arginine. Arginine competes for the same transporter in the blood–brain barrier, reducing the amount of lysine that enters the brain, hence reducing its toxic metabolites [22]. If a patient persistently refuses or vomits the emergency drinks, glucose, fluids and electrolytes should be given intravenously; the lysine‐free amino acid mixture may be given as a slow nasogastric infusion.

Regular carnitine supplements are recommended from the time of diagnosis in GA 1 to prevent depletion, the dose being doubled during acute illness [22]. Fever should be managed with antipyretics as there is evidence that it increases the risk of neurological damage. The risk of acute encephalopathy is low after 6 years of age, and emergency management may not be needed but currently it is still recommended [22].

## 5 Hyperammonaemic Encephalopathy

Hyperammonaemia is the classic acute problem caused by intoxication disorders. It is particularly prominent in urea cycle disorders and branched‐chain organic acidaemias, though it also occurs in other metabolic disorders, such as fatty acid oxidation disorders. Although ammonia has adverse effects on other organs, such as the liver, it is most toxic to the brain, where multiple cellular consequences lead to acute encephalopathy and long‐term damage [23].

**FIGURE 2:**
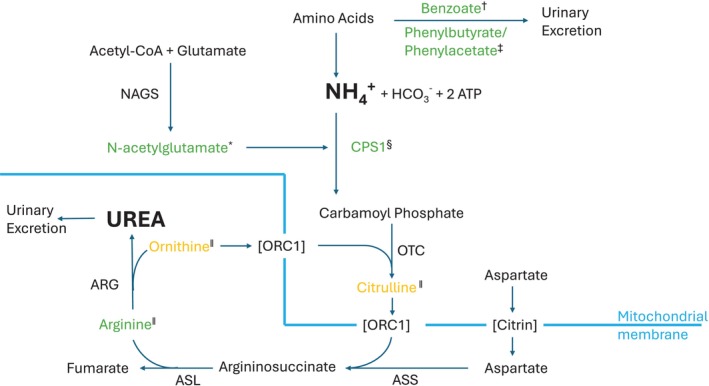
Simplified pathways of ammonia metabolism and treatment. Chemicals in green are involved in emergency treatment that is discussed in this article. Chemicals in yellow are used for the treatment of some conditions but not in emergencies and not discussed here. *Carglumic acid is a therapeutic analogue of N‐acetylglutamate. ^†^Benzoate binds to and removes glycine. ^‡^Phenylacetate binds to and removes glutamine. ^§^CPS1 is upregulated by CMP‐CPS‐001 (see text). ^‖^These amino acids are given to treat various disorders. ARG, arginase; ASL, argininosuccinate lyase; ASS, argininosuccinate synthetase; CPS1, carbamoyl‐phosphate synthetase 1; HCO_3_^−^, bicarbonate; NAGS, N‐acetylglutamate synthase; ORC1 ornithine/citrulline antiporter; NH_4_^+^, ammonia; OTC, ornithine transcarbamylase.

**FIGURE 3:**
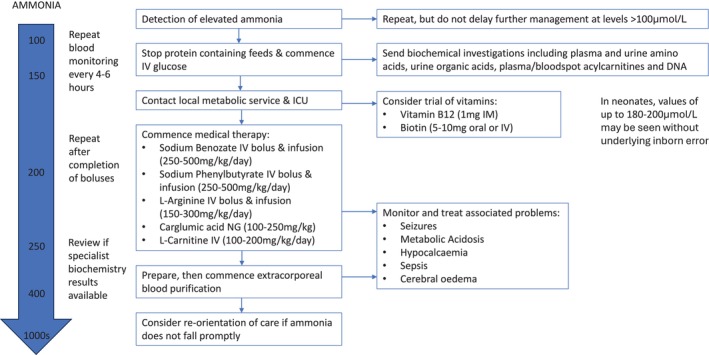
Summary of management for hyperammonaemia. The boluses of drugs are given over 90 min. IM, intramuscular; IV, intravenous; NG, nasogastric.

The pathways involved in hyperammonaemia are shown in Figure 2, and its management is summarised in Figure 3. Measures to prevent catabolism and reduce flux through the affected pathway are particularly important: glucose is given except in citrin deficiency, which is discussed below. In urea cycle disorders, the affected pathway can be bypassed, to an extent, using *nitrogen scavenging medications*. Phenylbutyrate/phenylacetate and benzoate achieve this by converting glutamine and glycine, respectively, to chemicals that are excreted in urine, reducing the plasma amino acid levels and the formation of ammonia. Infusions of both should be commenced (usually preceded by a priming infusion over 90 min) if the ammonia concentration exceeds 150 μmol/L [24, 25, 26]. Sodium benzoate can safely be used in patients with undiagnosed hyperammonaemia; its use in organic acidaemias has not caused problems [27], but carglumic acid is more effective (see below). There is more concern about giving sodium phenylbutyrate or phenylacetate to patients with organic acidaemias, as their plasma glutamine concentrations are likely to be low [27].

*L‐arginine* should also be given in urea cycle disorders (except arginase deficiency), as the defects prevent its synthesis; it is particularly effective at lowering ammonia acutely in arginosuccinate lyase deficiency [24, 25, 26]. L‐carnitine is often given to patients with undiagnosed hyperammonaemia because it is indicated in organic acidaemias, although it may possibly have a deleterious effect in fatty acid oxidation disorders [5, 26]. Vitamin B12 and biotin [24] may also be given, but few patients with severe hyperammonaemia respond.

*Carglumic acid* is an analogue of N‐acetylglutamate (NAG), which activates carbamoyl‐phosphate synthetase 1 (CPS1), the first step in the urea cycle (Figure 2). It is, therefore, the most effective treatment for hyperammonaemia in NAG synthase deficiency and in the branched‐chain organic acidaemias, where the defect leads to NAG synthase inhibition. Carglumic acid is also used for hyperammonaemia in fatty acid oxidation disorders, which is at least partly due to reduced NAG synthesis. For hyperammonaemia in severe carnitine‐acylcarnitine translocase deficiency, however, carglumic acid and nitrogen scavengers seem to confer limited benefit when added to high doses of glucose [28].

If the ammonia level exceeds 250 μmol/L, transfer may be needed for *extracorporeal blood purification*. Paediatric consensus guidelines for this have recently been published [29]. Peritoneal dialysis is much less effective and is only recommended if there are no alternatives. The method of extracorporeal blood purification will depend on what is available: continuous veno‐venous haemodiafiltration is preferred, but continuous veno‐venous haemofiltration can also be used. Intermittent haemodialysis lowers ammonia concentrations faster, but it may cause haemodynamic problems in neonates, and there is often rebound hyperammonaemia necessitating multiple sessions. Over recent years, the equipment has improved, particularly for babies [30]. The consensus guidelines recommend starting extracorporeal blood purification if the ammonia reaches 400 μmol/L and at a lower level if it is rising fast or causing neurological impairment [29]. Two recent studies, however, found that current practice does not improve the neurocognitive outcome in neonatal‐onset urea cycle disorders and has little effect on mortality [31, 32]. Starting at a lower ammonia concentration has been suggested, maybe as low as 180 μmol/L [33]. Adults develop cerebral oedema at lower ammonia levels, so extracorporeal blood purification is recommended at 200 μmol/L [5]. Neuroprotection with therapeutic hypothermia has been undertaken during dialysis [34] but further randomised data are needed to establish whether the benefits outweigh the difficulties [34, 35].

Sadly, outcomes are poor after severe hyperammonaemia, unless very brief, and it may be appropriate to re‐orientate care towards compassionate palliation [25]. Large series suggest that about 25% of patients with neonatal‐onset urea cycle disorders die during the presenting episode [36, 37]. Mortality during the presenting episode is about 40% for neonatal‐onset ornithine transcarbamylase (OTC) deficiency [32, 36, 38] and 8%–10% for later onset OTC deficiency [36, 38]. A review of neonatal studies in 2015 found no change in mortality over the preceding 3 decades [39]. Moreover, many patients who survive neonatal hyperammonaemia have cognitive impairment, which correlates with the severity of hyperammonaemia and its duration [33, 40, 41]. New and more effective treatments are needed, yet the most recent significant development was carglumic acid 15 years ago [42].

A few drugs currently being developed may help with acute hyperammonaemia in the future. CMP‐CPS‐001 is an antisense oligonucleotide which aims to upregulate CPS1 mRNA, increasing Carbamoyl Phosphate Synthetase production and the activity of the urea cycle (Figure 2). JNT‐517 is an inhibitor of a neutral amino acid transporter in the kidney and intestine, encoded by SLC6A19. Though defects of this gene can cause Hartnup disease, most patients have asymptomatic aminoaciduria, and a trial of JNT‐517 in phenylketonuria is underway [43]. SLC6A19 knockout improved the survival and biochemistry in OTC‐deficient mice [44], so it is possible that JNT‐517 may help patients with urea cycle disorders. Finally, glutaminase‐2 inhibitors are being developed as anticancer drugs [45]. AAV‐induced knockdown of Gls2, the gene encoding glutaminase‐2, reduced hyperammonaemia and increased survival in OTC‐deficient mice [46]. Glutaminase‐2 inhibitors may, therefore, have a role in hyperammonaemia.

## 6 Citrin Deficiency

Citrin deficiency is a rare cause of acute hyperammonaemia in adolescents or adults [47]; it differs from other causes of hyperammonaemia in being worsened, rather than improved, by giving glucose [48]. Citrin is an aspartate/glutamate antiporter found in the inner membrane of hepatic mitochondria (Figure 2). As well as carrying aspartate to the cytoplasm for use in the urea cycle, it is part of the malate‐aspartate shuttle, which moves reducing equivalents (produced by glycolysis) into mitochondria [48]. Giving glucose worsens the accumulation of reduced cofactors in the cytoplasm and exacerbates hyperammonaemia [49]. Emergency management is with intravenous sodium benzoate, sodium phenylbutyrate and arginine, and, if necessary, haemofiltration; energy is best provided as enteral medium‐chain triglycerides (MCT) [50]. The diagnosis is unlikely to be made acutely but may already be known, as many patients present in infancy with liver disease (discussed later) or in childhood with failure to thrive and unusual dietary preferences [47]. In the past, the prognosis was poor and liver transplantation was recommended, but outcomes with conservative treatment have been better since glucose has been avoided and MCT used.

## 7 Epileptic Encephalopathies

Inherited metabolic disorders are rare causes of epileptic encephalopathy. They present most often in the neonatal period, but even then, they account for fewer than 1 in 40 cases [51]. Some remain untreatable (e.g., glutaminase deficiency [52]) but others have specific treatment [53]. The best known are the *pyridoxine‐responsive epilepsies*, of which antiquitin deficiency is the commonest. Antiquitin encodes α‐aminoadipic semialdehyde dehydrogenase, which is involved in lysine degradation. Antiquitin deficiency leads to the accumulation of Δ^1^‐piperideine‐6‐carboxylate; this inactivates pyridoxal 5‐phosphate (PLP), which is a cofactor for around 70 reactions. PLP can be replenished by giving pyridoxine, and the International League Against Epilepsy recommends using this empirically in the treatment of refractory neonatal seizures [54]. Most patients respond to 100 mg pyridoxine, intravenously or enterally, but a few show a delayed response if given 30 mg/kg/day for 3 days. Seizures sometimes recur during febrile illnesses, despite long‐term treatment with pyridoxine and a low‐lysine diet. To reduce this risk, a consensus guideline recommends doubling the pyridoxine dose and providing sufficient calories to prevent catabolism during illnesses [55]. Folinic acid has also been recommended if seizures recur [56].

Pyridox(am)ine 5′‐phosphate oxidase (PNPO) is responsible for the synthesis of PLP from pyridoxine and for its recycling [57]. PNPO deficiency is clinically indistinguishable from antiquitin deficiency, except that most patients are born prematurely. Some respond to pyridoxine, but a trial of PLP should be considered if neonatal epileptic encephalopathy does not respond to pyridoxine or shows a partial response [58, 59]. Pyridoxine‐responsive seizures have also been reported in hyperprolinaemia type II. Pyrroline‐5‐carboxylate accumulates and inactivates PLP [60] by a similar reaction to that in antiquitin deficiency. Long‐term treatment with pyridoxine is recommended, though there was little evidence of benefit in one small series [61].

Seizures may also be a presenting feature for *biotinidase and holocarboxylase synthetase* (*HCS*) *deficiencies*. Severe biotinidase deficiency has predominantly neurological features and typically presents between 1 and 4 months of age [62]. In contrast, patients with HCS deficiency can present at any age from a few hours to mid‐childhood, usually with acidosis, which may be accompanied by hyperammonaemia [63]. Alopecia and rashes occur in both conditions. All patients with biotinidase deficiency respond to biotin, as do most with HCS deficiency, though higher doses may be needed [64]. Deafness and neurodevelopmental problems are very common in patients with biotinidase deficiency presenting clinically but can be prevented by newborn screening [65]. Most patients need no special treatment during acute illness if biotin is continued, but some with HCS deficiency require a high caloric intake to prevent decompensation, and insulin is reported to have helped one patient [66].

*Non‐ketotic hyperglycinaemia* (NKH) is caused by a deficiency of the glycine cleavage system [67]. Most patients present within hours or days of birth with poor feeding, hypotonia and lethargy, progressing to coma and, usually, apnoea. If ventilatory support is provided, spontaneous respiration resumes after about 10 days, but most patients have intractable epilepsy and make little developmental progress. A smaller number of patients present after 2 weeks of age with developmental delay and seizures. Treatment is primarily with sodium benzoate, which lowers glycine levels and is sometimes combined with dietary restriction of glycine and serine [68]. Glycine is an activator of NMDA glutamate receptors, and treatment with the NMDA receptor antagonists Dextromethorphan or Ketamine can also help [69]. Patients often require multiple anticonvulsants. The ketogenic diet can improve seizure control, as in other epilepsies, and has the additional benefit of lowering glycine levels by reducing its synthesis [70, 71].

NKH is classified into severe and attenuated phenotypes according to their outcome [72]. Patients with the severe phenotype show minimal developmental progress; some learn to smile and suck from a bottle, but most are tube fed by 6 months, with spastic quadriplegia, cortical blindness and intractable seizures [73]. Patients with the attenuated phenotype have little spasticity and seizures may resolve with sodium benzoate and dextromethorphan or with one anticonvulsant; developmental progress is variable—they can sit and often walk and understand language but have few words and severe hyperactivity. Given the bleak outlook for the severe phenotype, withdrawal of intensive care may be considered when patients are ventilator dependent. The phenotype cannot, however, be predicted reliably from the presentation: 15% of patients presenting in the first 2 weeks have the attenuated form, and 50% of those presenting between 2 weeks and 3 months have the severe form [74]. The phenotype can, however, be predicted from features such as neuroimaging and glycine concentrations: a severity score has been proposed by the International Working Group on Neurotransmitter Related Disorders [75, 76].

*Sulphite oxidase deficiency* (*iSOD*) *and molybdenum cofactor* (*MoCo*) *deficiency* both lead to sulfite intoxication and cause encephalopathy, with or without seizures, in the early neonatal period, after a symptom‐free interval of 24–72 h; a few atypical patients present later [77, 78]. Typical patients progress to intractable epilepsy and profound developmental delay [79]. The two disorders are clinically indistinguishable at presentation, but the plasma urate quickly falls to low or undetectable concentrations in MoCo deficiency, remaining normal for age in iSOD [77, 79]. This is relevant as treatment is now available for MoCo deficiency type A, caused by deficiency of the first enzyme involved in MoCo synthesis [80]. Fosdenopterin (cyclic pyranopterin monophosphate) is the product of the deficient enzyme, so its administration allows MoCo synthesis. Neuroimaging has shown that some damage in MoCo deficiency occurs prenatally [81]. Nevertheless, patients treated before the onset of severe encephalopathy have remained seizure‐free with near‐normal development [80]. As delay leads to profound handicap, treatment should be started without genetic results and stopped in patients found to have MoCo deficiency types B or C.

## 8 Stroke Like Episodes, Strokes and Other Thromboembolic Events

In stroke‐like episodes (SLEs), sometimes called ‘metabolic strokes’, an acute metabolic derangement ‘leads to the rapid onset of prolonged central neurological deficits in the absence of vessel occlusion or rupture’ [82]. SLEs are particularly associated with primary mitochondrial disorders, especially mitochondrial myopathy, encephalopathy, lactic acidosis and stroke‐like episodes (MELAS) syndrome. They can also occur in acute intoxication disorders [82, 83], including urea cycle disorders and organic acidaemias, even after liver transplantation [84]. The initial work‐up is likely to include neuroimaging, which can exclude a primary vascular cause [85].

**FIGURE 4:**
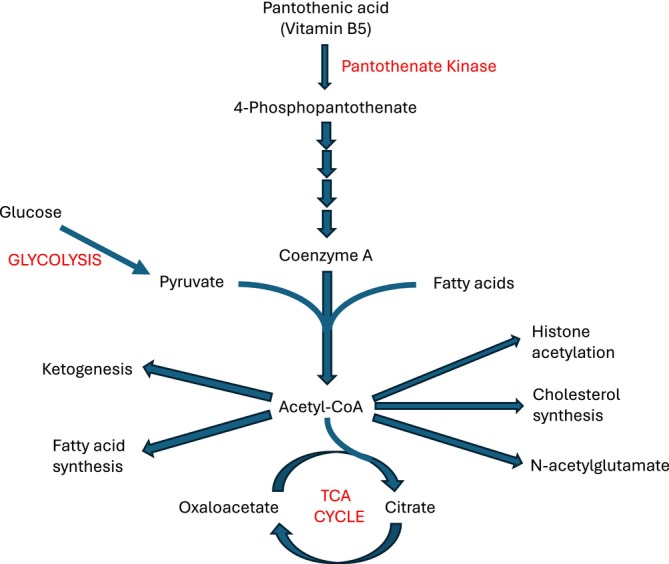
Simplified pathways of coenzyme A synthesis and metabolism. TCA, tricarboxylic acid.

Management of SLEs is currently supportive, with hydration, prevention of catabolism and correction of any metabolic derangement [83]. The pathogenesis in organic acidaemias probably involves mitochondrial dysfunction, for which there are multiple possible mechanisms [86], including sequestration of coenzyme A (CoA). The pathways involved in CoA metabolism are summarised in Figure 4. In a mouse model of propionic acidaemia (PA), an activator of pantothenate kinase (BBP‐671) restored CoA levels and improved the metabolic profile and survival [87]. As this activator crosses the blood–brain barrier [88] it may ameliorate the neurological complications of organic acidaemias. Unfortunately, a clinical trial of this compound in PA, and methylmalonic aciduria has recently closed because of slow recruitment [89].

Strokes and other thromboembolic complications are prominent in disorders with markedly increased homocysteine concentrations, namely cystathionine beta‐synthetase (CBS) deficiency [90] and remethylation disorders, such as Cobalamin C and E defects and MTHFR deficiency [91]. The risk is mainly for venous thromboses, and the strokes in young patients are often because of cerebral venous sinus thrombosis [92], though they can be arterial [93]. Deep venous thromboses are a common presenting feature in patients with pyridoxine‐responsive CBS deficiency [90, 94]. Though the pathogenesis is still poorly understood [95] the risk of thromboses is related to the plasma total homocysteine concentration and can be eliminated by good control.

The emergency management of thromboembolism is the same in the homocystinurias as in other patients, with anticoagulants and adequate hydration. Patients with cerebral venous sinus thrombosis may also require anticonvulsants and intracerebral pressure monitoring. Thrombolysis may be appropriate for high‐risk pulmonary emboli. Homocysteine levels are generally very high and should be lowered with pyridoxine, dietary management, betaine and/or hydroxocobalamin, depending on the precise diagnosis.

## 9 Liver Failure

*Galactosaemia* (GALT deficiency) and hereditary fructose intolerance (aldolase B deficiency) are both disorders of carbohydrate metabolism [96] and among the first diagnoses that should be excluded in any child presenting with acute liver disease. Classical galactosaemia usually presents in the early neonatal period with poor feeding and jaundice, rapidly followed by other problems. It is the commonest inherited metabolic cause of liver failure in children [97]. Breast milk and standard infant formulas need to be stopped immediately when the diagnosis is suspected, pending confirmation by genetic testing or enzyme analysis [98]. A galactose‐free formula is substituted; traditionally, these have been soy‐based but alternatives are available [99]. Antibiotics should be started routinely as patients have a propensity to Gram‐negative sepsis at this stage, with positive blood cultures in over half the cases [96]. Liver function almost always recovers, but occasionally urgent liver transplantation is needed. Dietary changes do not prevent the long‐term complications of neurodevelopmental delay and ovarian failure [96]. Other therapeutic strategies are being developed to address these issues, but they are not relevant in the acute situation [100].

*Hereditary fructose intolerance* may present in infancy or later, sometimes even in adults. It is seldom seen before weaning because fructose is absent from breast milk and most infant formulas [101], though there are exceptions [102]. Initial features may be hypoglycaemia, vomiting or abdominal pain, with prolonged exposure leading to acute liver failure and renal tubulopathy [103]. Biochemical clues include lactic acidosis, hypermagnesemia, hyperuricaemia and hypophosphataemia; transferrin isoelectric focusing, if performed, may show abnormal glycosylation [104]. A careful dietary history can raise suspicion and allow management to be started before the diagnosis is confirmed by molecular genetics. Patients are treated by excluding fructose, sucrose and sorbitol from the diet, with supportive management whilst the liver and kidney injuries resolve [1]. The prognosis is excellent with normal development and life expectancy.

Patients with *hereditary tyrosinaemia type 1* (HT1, fumarylacetoacetate hydrolase deficiency) present with liver disease at any age from 2 weeks to adulthood, most commonly with acute liver failure in the first 6 months of life [105]. Patients may also develop renal tubulopathy and neurological crises with pain and weakness, which can progress to respiratory failure [105]. The emergency management is with support (e.g., antibiotics, correction of severe coagulopathy and ventilation if necessary) and nitisinone (NTBC). This blocks the second step in tyrosine degradation before the toxic metabolites are formed [106]. Most patients improve rapidly, though a few require urgent liver transplantation. Outcomes are best if NTBC is started pre‐symptomatically [106], which also reduces the long‐term risk of hepatocellular carcinoma. Succinylacetone accumulates in HT1, and its measurement in bloodspots by tandem mass spectrometry is sensitive and specific enough for newborn screening [107] but one missed case has been reported [108].

*Citrin deficiency* has been considered earlier in this article as a cause of hyperammonaemia in adults. It can also present in the first year of life with prolonged cholestatic jaundice, transaminitis and coagulopathy, when it is called neonatal intrahepatic cholestasis caused by citrin deficiency (NICCD) [109]. Biochemical clues may include elevated citrulline and an elevated threonine/serine ratio [49]. Although liver failure is often transient, in rare cases it can require transplantation [110]. As in adults with hyperammonaemia, treatment with MCT helps, often leading to improved liver function within a week [48]. It is worth noting that galactose may be present in blood and urine from these patients, and a galactose‐free formula is recommended [48].

*Wilson disease* is caused by impaired biliary copper excretion and its toxic accumulation, particularly in the liver and brain [111]. Neurological symptoms include dysarthria, dystonia, ataxia, tremor and psychiatric problems, usually starting between 8 and 40 years of age. Hepatic presentations are typically between 3 and 20 years of age and range from asymptomatic rises in transaminases to cirrhosis; 3%–5% of all patients present in acute liver failure, when diagnosis can be challenging [111, 112]. Treatment is with copper chelators (penicillamine or trientine) or zinc; chelators have more side effects but remove copper faster and should be used for liver disease, as this can progress rapidly. Patients with acute liver failure and encephalopathy require urgent transplantation [111]. Some patients with acute liver failure but no encephalopathy can be managed with copper chelation; a scoring system has been devised to predict who will need a liver transplant [113]. Further therapeutic options are needed but, disappointingly, a Phase 3 study of tetrathiomolybdate, a novel chelator, was recently terminated by the sponsor [114].

## 10 Cardiomyopathy and Arrhythmias

Cardiomyopathy and arrhythmias are rarer in intoxication‐type disorders than in disorders of energy metabolism, but they are seen in several organic acidaemias, particularly PA [115]. The cardiomyopathy in *PA* is usually dilated and may develop slowly or acutely during an episode of decompensation. Any accompanying metabolic disturbance must be treated, but the cardiomyopathy generally persists. Cardiomyopathy is reported to have responded to coenzyme Q10 in one patient [116] but the consensus guideline does not recommend its use [84]. In some patients, cardiomyopathy has resolved after liver transplantation [117] but it can recur [118]. Even without cardiomyopathy, patients with PA often have a prolonged QTc interval, and they are at risk of ventricular arrhythmias [119]. The emergency management of the arrhythmias is the same as in patients without PA, but if monitoring shows a prolonged QTc interval, precautions can be taken to reduce the risks.

Cardiomyopathy has also been reported in methylmalonic aciduria (rarely), D‐2‐hydroxyglutaric aciduria type II and malonic aciduria. Patients with malonic aciduria may benefit from treatment with carnitine and a diet supplemented with MCT. This is because the accumulating malonyl‐CoA inhibits carnitine palmitoyl transferase I, preventing fatty acids from entering mitochondria [120]. Medium‐chain fatty acids can enter mitochondria independent of carnitine and be oxidised.

The pathogenesis of *TANGO2 deficiency disorder* is uncertain, and it may not be an intoxication‐type disorder; we mention it because specific management has recently been reported. Patients have developmental delay and neurological problems, but many present acutely with cardiomyopathy, QTc prolongation and arrhythmias (mostly torsade de pointes), as well as hyperammonaemia, hypoglycaemia and rhabdomyolysis. Arrhythmias are the commonest cause of death [121]. TANGO2 deficient Drosophila have similar problems, which improved after they were given pantothenic acid (vitamin B5), a precursor of CoA [122] (Figure 4). A natural history study subsequently found that patients supplemented with vitamin B‐complex had not suffered metabolic crises [121]. Vitamin B‐complex is now recommended for all patients, especially during crises [123]. Given its safety, treatment should be considered in undiagnosed patients presenting acutely with compatible symptoms.

## 11 Rhabdomyolysis

Rhabdomyolysis has various genetic causes, including defects of energy metabolism, such as fatty acid oxidation disorders [124]. It is also seen in TANGO2 deficiency disorder (discussed above) and *lipin 1 deficiency*, which causes particularly severe episodes with a mortality of up to one third [125]. Lipin 1 deficiency is probably not an intoxication disorder—the pathogenesis may involve inflammation and an energy defect [126]—but we mention it because emergency management has recently been reported. Corticosteroids administered acutely reduce the duration of severe rhabdomyolysis and improve survival. In the original report, none of the 10 patients treated with steroids died, whereas all 4 patients who did not receive steroids died, despite appropriate measures to correct hyperkalaemia [126]. In view of the risk and prevalence of lipin 1 deficiency, there is a case for giving steroids to all young children with rhabdomyolysis.

## 12 Conclusions

Intoxication‐type metabolic disorders have been recognised for longer than most other inborn errors, and their pathogenesis is relatively well understood. This has allowed the development of logical treatment strategies, usually aiming to reduce levels of the accumulating chemical. For many of the disorders, there are now evidence‐based guidelines; these have promoted research [127] as well as improving management, and we have referred to them repeatedly in this review.

Unfortunately, outcomes are often disappointing, and there has been little improvement for several decades. Few new developments relate to emergency management, but there has been some progress, such as the treatment of MoCo deficiency type A with Fosdenopterin. This and many other emergency treatments need to be started very quickly. Newborn screening has facilitated this for some disorders. For others, treatment depends on identifying the disorder promptly, which is best achieved with biochemical tests; these must not be displaced by ‘rapid’ whole genome sequencing. Most non‐specialists are aware of the potential for life‐saving management in intoxication‐type disorders, but improved outcomes still depend on earlier recognition and treatment.

## Author Contributions

J. Dexter Tarr and Andrew A. M. Morris both contributed to the review of relevant materials and the writing of this article.

## Disclosure

Andrew A.M. Morris accepts full responsibility for the work and/or the conduct of the study, had access to the data, and controlled the decision to publish.

## Ethics Statement

The authors have nothing to report.

## Consent

The authors have nothing to report.

## Conflicts of Interest

J. Dexter Tarr received financial reimbursement from Sanofi S.A. to attend the SSIEM 2024 and a speaker fee for the arrangement of education following this. Andrew A.M. Morris has received honoraria for lectures from Travere and Nutricia Metabolics and sat on an advisory board for Evinacumab (Ultragenyx), all unrelated to this work.
